# Germinal Center B-Cells Resist Transformation by *Kras* Independently of Tumor Suppressor *Arf*

**DOI:** 10.1371/journal.pone.0067941

**Published:** 2013-06-25

**Authors:** Chelsea D. Mullins, Mack Y. Su, Vishwanathan Hucthagowder, Liang Chu, Lan Lu, Shashikant Kulkarni, Deborah Novack, Ravi Vij, Michael H. Tomasson

**Affiliations:** 1 Department of Internal Medicine, Washington University School of Medicine, Saint Louis, Missouri, United States of America; 2 Department of Pathology & Immunology, Washington University School of Medicine, Saint Louis, Missouri, United States of America; 3 Department of Genetics, Washington University School of Medicine, Saint Louis, Missouri, United States of America; University of Michigan School of Medicine, United States of America

## Abstract

Activating mutations in Ras (N- and K-) are the most common point mutations found in patients with multiple myeloma (MM) and are associated with poor clinical outcome. We sought to directly examine the role of Ras activation in MM pathogenesis and used two different tissue-specific Cre recombinase mouse lines (*Cγ1-Cre* and *AID-Cre*), to generate mice with mutant Kras (*Kras^G12D^*) activated specifically in germinal center B-cells. We also generated mice with activation of the *Kras^G12D^* allele in a tumor-prone Arf-null genetic background. Surprisingly, we observed no significant disruption in B-cell homeostasis in any of these models by serum immunoglobulin ELISA, SPEP, flow cytometry and histological examination. We observed development of non-overlapping tumor types due to off-target Cre expression, but despite successful recombination in germinal center and later B-cell populations, we observed no B-cell phenotype. Together, these data demonstrate that Ras activation is not sufficient to transform primary germinal center B-cells, even in an Arf-null context, and that the temporal order of mutation acquisition may be critical for myeloma development. Specific pathways, yet to be identified, are required before Kras can contribute to the development of MM.

## Introduction

Multiple myeloma (MM) is an incurable malignancy of antibody-secreting plasma B-cells, whose etiology remains poorly understood. Mutations in Ras genes, encoding key proteins regulating cell growth, differentiation and survival, occur commonly in MM with a prevalence of 20–39% [1]–[3]. Indeed, using a targeted sequencing approach to screen highly expressed tyrosine kinase and cytokine signaling genes in primary human patient myeloma, we previously identified mutations at codon 12 and 61 in N- and KRAS as being the only recurrent variation in our sample set [4]. Recent genome sequencing efforts also found Ras mutations to be the most common single nucleotide variant (SNV) in MM [4], suggesting that Ras activation is an important event in MM pathogenesis. The somatic SNVs found most frequently in MM are gain-of-function mutations in Ras oncogenes (Kras and Nras), causing constitutive activation of the Ras protein [5].

Despite the genomic evidence for Ras pathogenesis, the functional role of Ras activation in MM has not previously been tested. This issue is not trivial as the induction of neoplasia by Ras activation is highly dependent on cellular context [6]. Understanding the effects of Ras activation in mature B-cells will allow us to better define the downstream pathways critical for development of MM. Moreoever, pharmaceutical approaches to target cancers with mutant Ras are underway [7]–[10], and a pre-clinical model faithfully replicating Ras-driven myeloma would be critical in evaluating the therapeutic potential of these agents in myeloma.

Post-germinal center (GC) B-cells are strongly implicated as the cell of origin in MM by demonstration of stable immunoglobulin (Ig) switch clonotypes over the course of disease [11], [12]. To test if expression of oncogenic Ras in GC B-cells was sufficient to induce myeloma, we utilized transgenic mice harboring a constitutively active Kras (G12D mutation) knocked-in to the endogenous Kras locus and flanked by a Lox-Stop-Lox cassette [13]. The Kras mouse model has been successfully used in several labs in developing cancer models [14], [15] [13], [16]. These mice were crossed with two different mature B cell-specific Cre recombinase (Cre) mouse strains (*Cγ1-Cre* and *AID-Cre*) to definitively test the effects of Ras activation in post-GC B-cells, including downstream memory B and plasma cells [17], [18]. As Ras activation can induce cellular senescence [19] and often requires cooperating mutations to induce transformation, so we also generated a strain of triple transgenic mice by crossing *Kras^G12D^* mice with mice null for the P19^ARF^ tumor-suppressor gene (Arf ^−/−^) [20]. *Arf* (P14^ARF^ in humans) is a potent tumor suppressor gene that cooperates with Ras activation in cellular transformation and carcinogenesis [21], [22]. In patients with myeloma, the P14/P16 locus is methylated in 42% [23], although the biological significance of this epigenetic modification is contested [24].

Surprisingly, in these settings we found B-cell development to be only subtly perturbed, even in the setting of *Arf* deficiency. Conversely, mice frequently developed tumors harboring Cre-recombined *Ras* alleles in non-B-cell tissues due to small amounts of off-target Cre expression. These data demonstrate that post-GC B-cells are resistant to transformation by mutations that are strongly oncogenic in other cellular contexts and that Ras activation must likely cooperate with tissue-specific mutations or epigenetic events to induce myeloma.

## Results

### *Cγ1-Cre Kras^G12^* ^D^ Mice Develop Thymic Lymphomas and Lung Adenomas but not Myeloma

**Figure 1:**
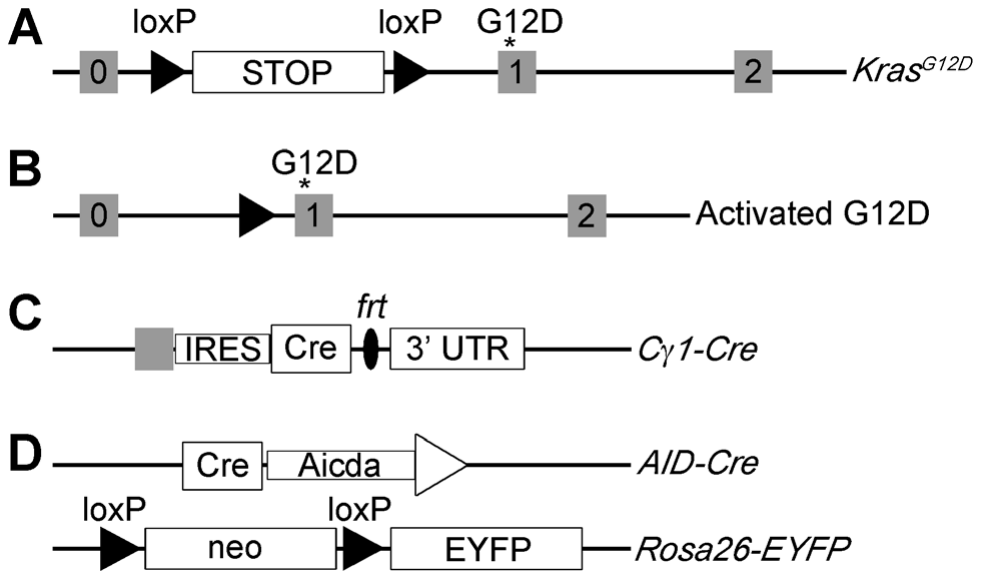
Schematic of alleles used in generating transgenic mice. **A)** Floxed *Kras* allele with exons 0, 1, and 2, under the endogenous Kras locus. Asterisk represents G12D mutation in exon 1. **B)** Excision of the stop cassette of the *Kras* allele by Cre recombinase allows the G12D mutation to activate. **C)** The Cre-coding sequence is knocked in downstream of the last coding exon of the Cγ1 locus. Expression of Cre recombinase is induced by transcription of the Ig γ1 constant region. **D)** After the floxed neomycin gene is deleted by Cre-mediation, the YFP is expressed alongside AID-expressing B cells.

To examine the effect of Kras in plasma cells, we generated double transgenic mice. In *Kras^G12^* ^D^ mice, the G12D mutation is knocked-in to the endogenous Kras locus, upstream of the Lox-Stop-Lox cassette (**Figure 1A,B** **)**. *Kras^G12^* ^D^ mice were crossed with mice expressing Cre recombinase (Cre) under control of the Ig heavy chain locus (*Cγ1-Cre*) reported to express Cre selectively in a subset of germinal center B-cells (**Figure 1C** **)**.

**Figure 2:**
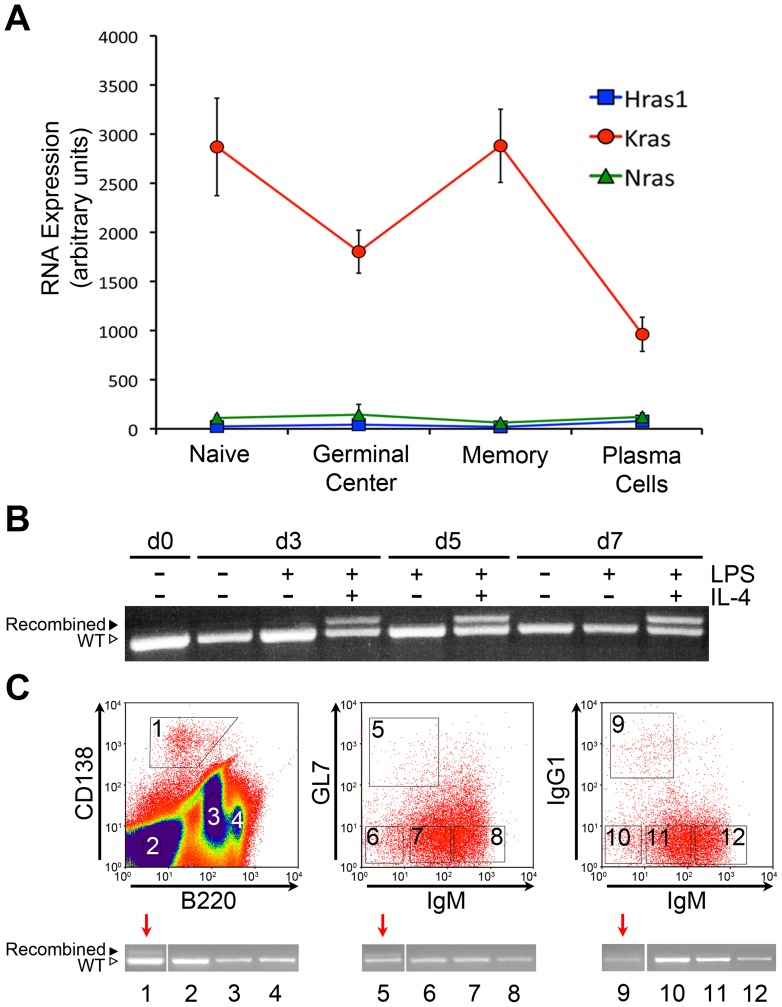
*Kras* expression in B-cell subsets and tissue-specific recombination in *Cγ1-Cre Kras^G12D^* mice. **A)** Expression of Ras genes by microarray in primary mature B-cell subsets; naïve splenic B-cells, germinal center B-cells, memory B-cells, and plasma cells. **B)** Successful Lox-Stop-Lox excision from Kras locus in B cells of *Cγ1-Cre Kras^G12D^* mice following class switch recombination. PCR of *Kras^G12D^* allele in B-cells of *Cγ1-Cre Kras^G12D^* mice stimulated to undergo class switch recombination ex vivo. Naïve splenic B-cells were stimulated to undergo class switch recombination with lipopolysaccharide (LPS) alone or LPS plus interleukin-4 (IL-4). Successful recombination was observed upon switch to IgG1 induced by LPS plus IL-4. **C)** Fluorescence activated cell sorting (FACS) isolation of mature B-cell subsets directly from *Cγ1-Cre Kras^G12D^* mice. In the first panel, bone marrow mononuclear cells; second and third panels, splenic mononuclear cells, both panels gated for B220^+^. Red arrows indicate lanes with detectable recombination. Recombination was low but detectable in bone marrow plasma cells (CD138^+^/B220^low^, lane 1); germinal center B-cells (B220^+^/GL7^+^/IgM^low^, lane 5) and IgG1 class switched splenic B-cells (B220^+^/IgM^−/^IgG1^+^, lane 9).

We first confirmed that wild type Kras is strongly expressed in murine B-lineage cells; naïve splenic B-cells, germinal center B-cells, memory B-cells and plasma cells from C57BL/6 mice (**Figure 2A**) [25]. As expected, Cre-mediated excision of the *Kras* allele stop cassette was robust and specific to B-lineage cells undergoing class-switch recombination *in vitro* (**Figure 2B** **and Figure S1**). We also confirmed Cre-recombination *in vivo* in mature B-cell populations isolated from *Cγ1-Cre Kras^G12^* ^D^ mice by fluorescence associated cell sorting (FACS). Splenic germinal center B-cells (B220^+^/IgM^−/^GL7^+^) and class switched memory/plasma cells (IgG1^+^) demonstrated clear, albeit low-level recombination, as did bone marrow plasma cells (B220^lo^/CD138^+^, **Figure 2C**).

**Figure 3:**
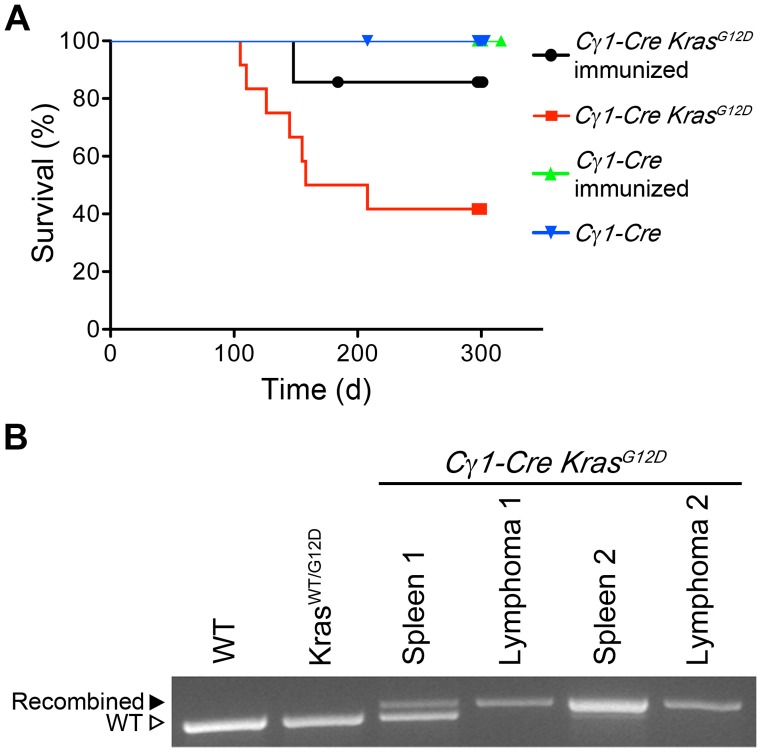
Development of T-cell lymphomas in*Cγ1-Cre Kras^G12D^* mice. **A)** Kaplan-Meier survival curves of aged *Cγ1-Cre Kras^G12D^* mice and *Cγ1-Cre* control mice cohorts. Naïve (unimmunized) *Cγ1-Cre Kras^G12D^* mice (n = 12) developed fatal T-cell lymphomas with a median latency of 125 days. Lung tumors were found incidentally at autopsy in both immunized *Cγ1-Cre Kras^G12D^* (n = 7) and naïve *Cγ1-Cre Kras^G12D^* mice, while naïve and immunized *Cγ1-Cre* were healthy for the duration and had no lung adenomas at autopsy. **B)** PCR detection of *Kras^G12D^* allele recombination in naïve *Cγ1-Cre Kras^G12D^* mice with T-cell lymphomas. Recombination is detectable in unfractionated mononuclear splenic cells, consistent with infiltration of spleen by lymphoma cells. Recombination with loss of wild-type allele observed in unfractionated cells isolated from thymic tumor tissue. Results from two affected mice are shown.

We aged *Cγ1-Cre Kras^G12^* ^D^ mice, both naïve and immunized with chicken gamma globulin to expand plasma cells, to monitor the development of disease. After 100 days, 58% (n = 12) of naïve mice developed weight loss, ruffled fur and shortness of breath and were found on necropsy to have thoracic cavity tumors. Unexpectedly, these tumors were T-lymphoblastic in phenotype (CD4^+^CD8^+^) by flow cytometry (**Figure S2A**). Additionally, 42% (n = 12) of naïve *Cγ1-Cre Kras^G12^* ^D^ mice and 66% (n = 7) CGG-immunized *Cγ1-Cre Kras^G12^* ^D^ mice were found to have lung nodules at autopsy (300 day endpoint). Sections of lung from immunized *Cγ1-Cre Kras^G12^* ^D^ show well-demarcated nodules composed mostly of sheets of bronchial epithelial cells and some “signet ring” cells with bland nuclear features and absence of mitotic figures consistent with adenomas or low-grade adenocarcinomas **(Figure S2B–E).** Tissue from lung tumors in two independent *Cγ1-Cre Kras^G12^* ^D^ mice shows partial recombination of the *Kras* allele **(Figure S2F).** The immunized and unimmunized negative control *Cγ1-Cre* mice showed no evidence of disease (**Figure 3A**). Tissue from T-cell lymphomas found in two separate unimmunized *Cγ1-Cre Kras^G12^* ^D^ mice showed complete *Kras* allele recombination, suggestive of loss of the wild-type allele, whereas spleen showed a partial recombination pattern consistent with infiltration of the spleen with these same cells (**Figure 3B**). Despite extensive analysis, no B-lineage oncogenic transformation was observed in any *Cγ1-Cre Kras^G12D^* mice. B-cell subsets in spleen and bone marrow and serum immunoglobulin levels were all normal (data not shown). Taken together, these data suggest that *Kras^G12D^* allele activation in germinal center B-cells failed to perturb B-cell homeostasis in *Cγ1-Cre Kras^G12^* ^D^ mice.

### *AID-Cre-YFP Kras^G12^* ^D^ Mice Develop Focal Epidermal Papillomas

Noting the low level of *in vivo* recombination in *Cγ1-Cre Kras^G12^* ^D^ mice (**Figure 2C**), and the lack of appreciable B- or plasma cell phenotype, we generated a second strain of mice using an independent tissue specific Cre allele. We crossed the *Kras^G12D^* mice with mice expressing Cre recombinase under the control of the activation-induced cytosine deaminase (AID) gene (**Figure 1D**). AID is expressed with exquisite specificity in B-cells undergoing the germinal center reaction where it mediates class switch recombination and somatic hypermutation. To facilitate our analysis, this strain of mice also included the Rosa26-EYFP reporter allele, which allowed us to effectively track B-cells where recombination had occurred (*AID-Cre-YFP Kras^G12D^*). Upon cre-mediated recombination, YFP marks cells where *Kras^G12D^* is also expressed. In an attempt to stimulate malignant B-cell transformation in *AID-Cre-YFP Kras^G12D^* mice, vitamin D deficient chow and/or sub-lethal radiation was given to cohorts of mice after immunization.

**Figure 4:**
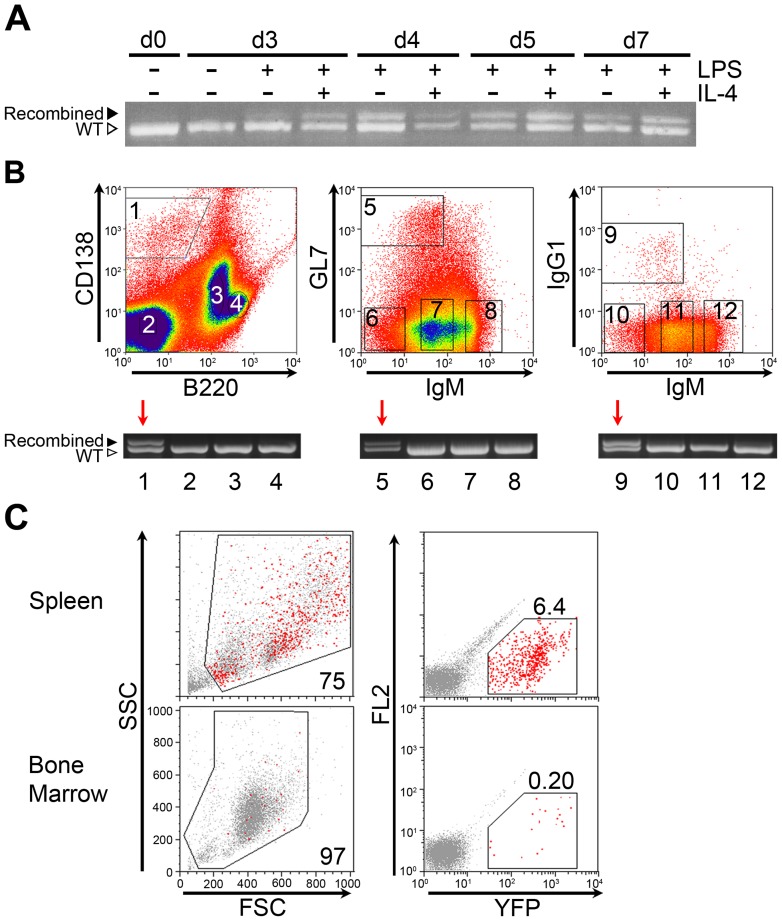
Efficient tissue specific recombination of*Kras* in class switched B cells of *AID-Cre-YFP Kras^G12D^* mice. **A)** PCR of *Kras^G12D^* allele in B-cells of *AID-Cre-YFP Kras^G12^* ^D^ mice stimulated to undergo class switch recombination *ex vivo*. Splenic B-cells were stimulated to undergo class switch recombination with lipopolysaccharide (LPS) alone or LPS plus interleukin-4 (IL-4). In contrast to *Cγ1-Cre Kras^G12D^* mice in Figure 2B, recombination was seen following stimulation with LPS+IL-4 or with LPS alone. **B)** FACS-purification of mature B-cell subsets from *AID-Cre-YFP Kras^G12^* ^D^ mice and detection of recombination by PCR. High-levels of Cre-mediated recombination in B220^lo^/CD138+ bone marrow plasma cells (lane 1), B220+/IgM−/GL7+ splenic germinal center B-cells (lane 5) and B220+/IgM−/IgG1+ class switched memory B-cell populations (lane 9) in *AID-Cre-YFP Kras^G12^* ^D^ mice. **C)** Detection of Cre-activated YFP reporter in cells isolated from spleen and bone marrow of *AID-Cre-YFP Kras^G12^* ^D^ mice given radiation and vitamin D deficient chow. Recombined, YFP-positive cells are plentiful in spleen (6.4%) but rare in the bone marrow (0.20%). Experiment was repeated with three mice and a representative example is shown.

Robust *Kras^G12D^* allele recombination was induced in *AID-Cre-YFP Kras^G12^* ^D^ splenic B-cells undergoing plasmacytic differentiation and class switch recombination *ex vivo* (**Figure 4A**). In contrast to the weak levels of *in vivo* recombination observed in *Cγ1-Cre Kras^G12D^* mice, germinal center splenocyte populations and post germinal center cells isolated from *AID-Cre-YFP Kras^G12^* ^D^ mice showed robust Cre-mediated recombination at both the *Kras^G12D^* locus (**Figure 4B**) and the YFP reporter in the spleen and to lesser extent in the bone marrow (**Figure 4C**).

**Figure 5:**
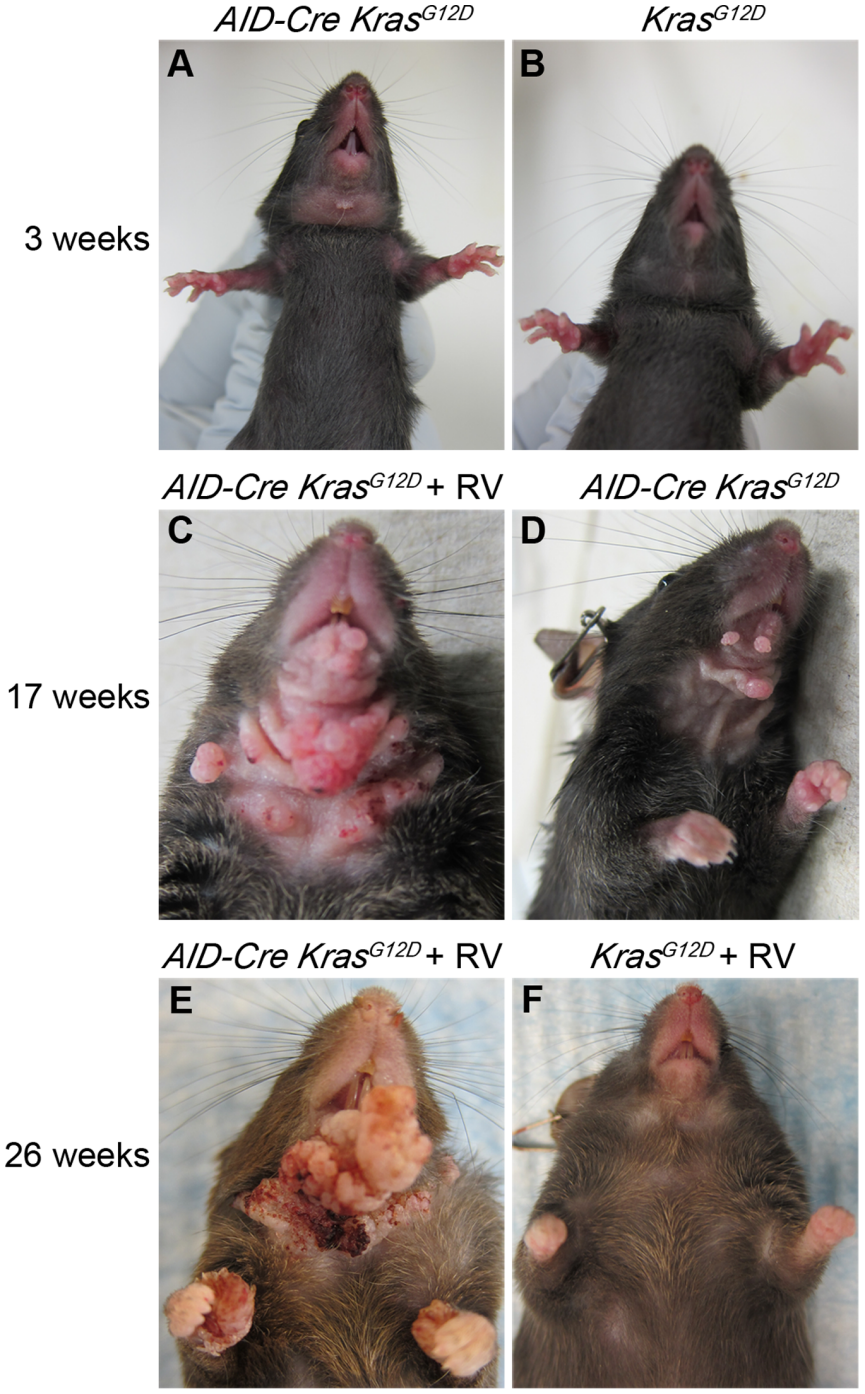
Gross appearance of cutaneous papillomas in*AID-Cre-YFP Kras^G12^* ^D^ mice is enhanced by tumor-promoting treatments. **A**) By 3 weeks of age, *AID-Cre-YFP Kras^G12^* ^D^ mice uniformly have hair loss and a single papilloma localized to the ventral neck; **B**) control *Kras^G12^* ^D^ mouse shows normal hair pattern and no papilloma; **C**) By 17 weeks, *AID-Cre-YFP Kras^G12^* ^D^ mice given radiation and vitamin D deficient chow (RV) had numerous fungating papillomas and more hair loss at the same site on the ventral neck; **D**) *AID-Cre-YFP Kras^G12^* ^D^ mice without tumor-promoting treatments also had progressive papillomas but much fewer and with less hair loss associated; **E**) *AID-Cre-YFP Kras^G12^* ^D^+RV mice aged to 26 weeks showed confluent fungating and ulcerated masses at the ventral neck with spread to paws; **F**) age-matched control *Kras^G12^* ^D^+RV mouse shows no similar signs.

**Figure 6:**
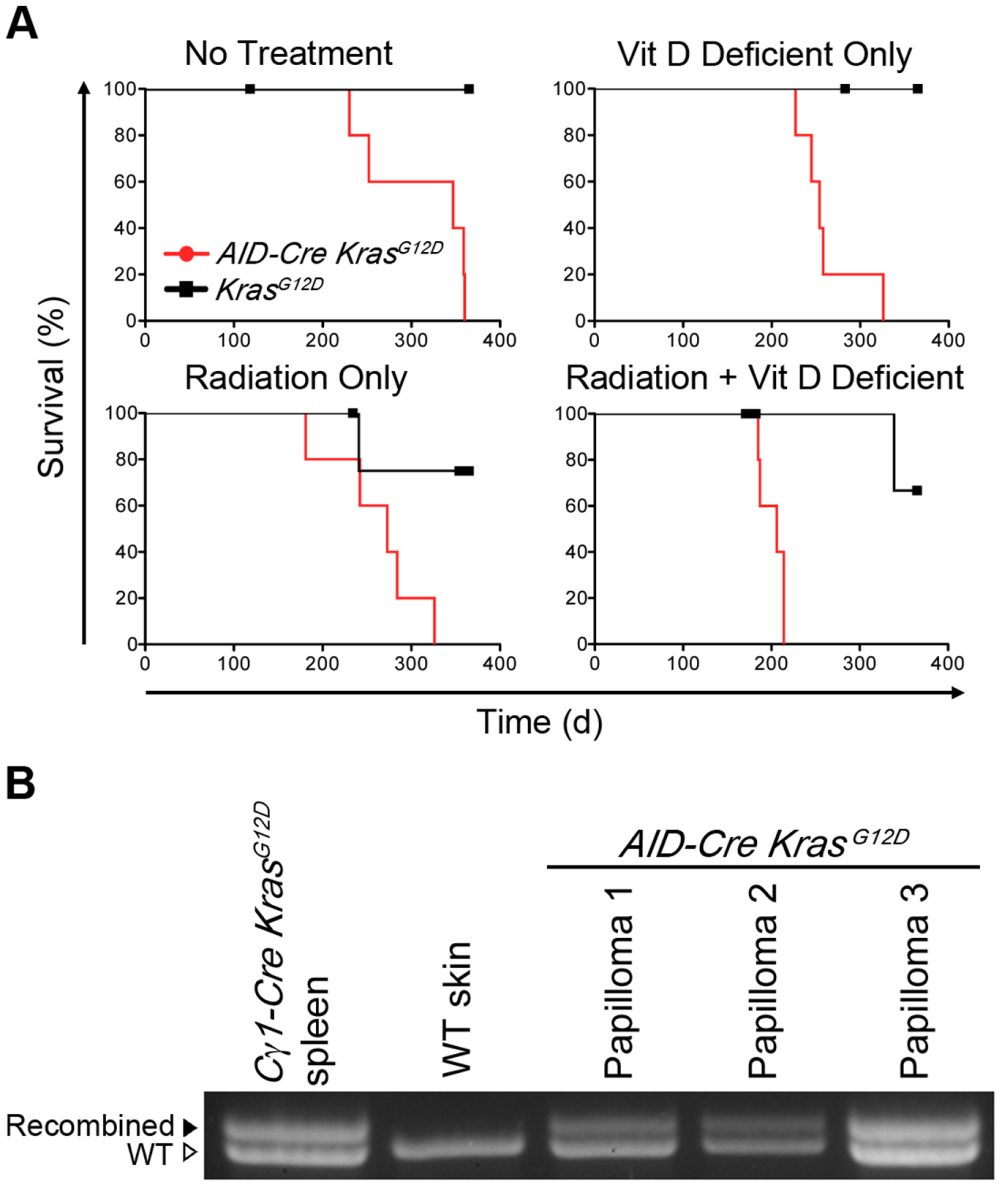
Cutaneous papillomas in*AID-Cre-YFP Kras^G12^* ^D^ mice and acceleration of lethality by tumor-promoting treatments. **A)** Kaplan-Meier survival curves of *AID-Cre-YFP Kras^G12^* ^D^ mice and control *Kras^G12^* ^D^ mice. Cohorts of *AID-Cre-YFP Kras^G12^* ^D^ and *Kras^G12^* ^D^ mice were subjected to vitamin D deficient chow continuously from 8.5 weeks of age or a single dose of sub-lethal gamma irradiation or given both. All *AID-Cre-YFP Kras^G12^* ^D^ mice developed progressive cutaneous papillomas that were made more extensive/aggressive with radiation or vitamin D deficiency. Mice were sacrificed when morbidity developed, defined by weight loss, unkempt coat, hunched posture, and lethargy. Each *AID-Cre-YFP Kras^G12^* ^D^ group had (n = 5) and developed papillomas, leading to infection, whereas every *Kras^G12^* ^D^ (n = 5) survived to day 352 endpoint. No B-cell phenotype was observed in any cohort. **B)** Cre-mediated recombination of *Kras* locus in DNA from papillomas was detected by PCR in three separate papilloma samples from *AID-Cre-YFP Kras^G12^* ^D^ mice. WT, wild-type control.

At 3 weeks of age, 100% (n = 20) *AID-Cre-YFP Kras^G12^* ^D^ mice lacked fur on the ventral neck and developed small growths, compared to control mice **(** **Figure 5A,B** **).** Radiation and Vitamin D deficient chow (RV) treatments increased the number and size of growths on *AID-Cre-YFP Kras^G12^* ^D^ mice as early as 17 weeks, compared to *AID-Cre-YFP Kras^G12^* ^D^ given neither (**Figure 5C,D**). By 26 weeks of age, all *AID-Cre-YFP Kras^G12D^* mice receiving both irradiation and vitamin D deficient chow (100%, n = 5) were hunched with ruffled fur and had infected lesions over the cutaneous growths with a median survival of 196 days **(** **Figure 5E, F** **).** *AID-Cre-YFP Kras^G12D^* mice with no treatment (besides immunization) at 26 weeks had an increase in the number of growths similar in appearance to that at 17 weeks. At 17 weeks, *Kras^G12D^* mice given both irradiation and vitamin D deficient chow appeared healthy without growths, similar to the 26 week timepoint (data not shown). All *AID-Cre-YFP Kras^G12D^* mice regardless of irradiation or vitamin deficient chow subsequently died or were sacrificed due to persistent skin infections associated with fungating skin lesions **(** **Figure 6A** **).** The cutaneous lesions were identified by histological examination to be benign papillomas (data not shown). Papillomas from 3 separate *AID-Cre-YFP Kras^G12^* ^D^ mice showed strong Cre-mediated recombination by PCR (**Figure 6B**). A small increase in total serum gamma region protein level achieved statistical significance in *AID-Cre-YFP Kras^G12^* ^D^ mice fed vitamin deficient chow (**Figure S3A**, middle panel), however the increase was not maintained over time, and mice treated with radiation, or no treatment at all had no significant changes in total serum gamma protein levels at any time point **(Figure S3A)**. Serum ELISA showed small changes among the antibody subtypes in *AID-Cre-YFP Kras^G12^* ^D^ mice, but no evidence of plasma cell transformation or any B-cell malignancy was found **(Figure S3B** and data not shown**)**.

### *AID-Cre-YFP Kras^G12^* ^D^ Arf^−/−^ Mice Develop Fatal Epidermal Papillomas and Derivative Carcinomas

**Figure 7:**
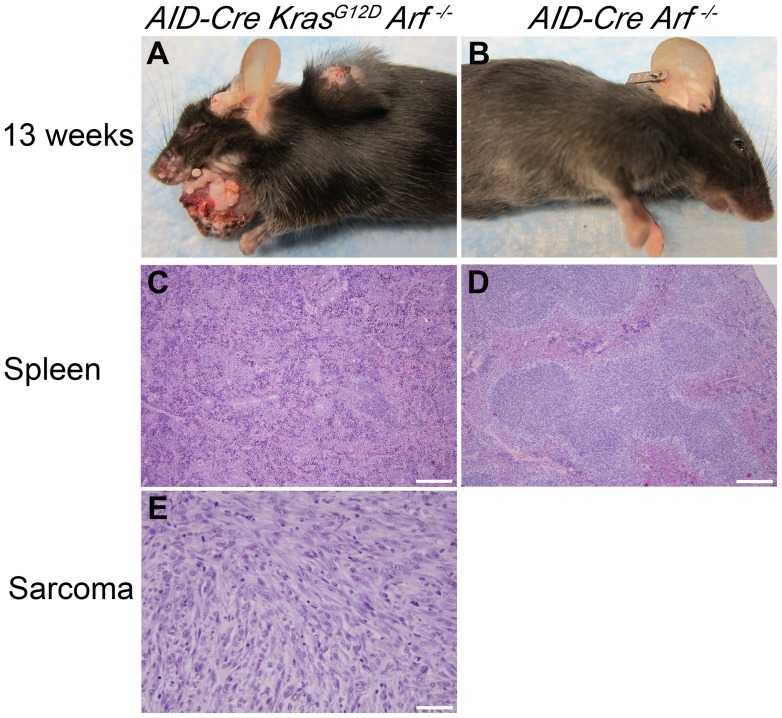
Aggressive papillomas and soft tissue sarcomas in*AID-Cre-YFP Kras^G12D^ Arf^−/−^* mice. **A)** Gross appearance of progressive tumors affecting an *AID-Cre-YFP Kras^G12D^ Arf ^−/−^* mouse at 13 weeks of age. Tumors progressed rapidly from smaller papillomas on the ventral neck. **B)** Control *AID-Cre-YFP Arf ^−/−^* control mouse at 13 weeks appears normal. Hematoxalin & eosin stains of **C)** spleen section from *AID-Cre-YFP Kras^G12D^ Arf ^−/−^* shows disruption of splenic architecture by inflammatory cells; **D)** spleen section from *AID-Cre-YFP Arf ^−/−^* control appears normal; and **E)** section of subcutaneous sarcoma from *AID-Cre-YFP Kras^G12D^ Arf ^−/−^* at 13 weeks shows spindle shaped cells consistent with soft tissue sarcoma. Original magnification, x10 (spleen); x40 (sarcoma). Scale bar: 200 um (spleen); 50 um (sarcoma). Tumor development was uniform in *AID-Cre-YFP Kras^G12D^ Arf ^−/−^* mice and did not require any tumor-promoting treatment. Representative images are shown.

We reasoned that a lack of a detectable B-cell phenotype in *Cγ1-Cre Kras^G12^* ^D^ and *AID-Cre-YFP Kras^G12^* ^D^ mice was most likely due to a requirement for a cooperating “second hit” to induce cellular transformation. Therefore, to test the effects of a second mutation known to cooperate with *Kras^G12D^*, we crossed *AID-Cre-YFP Kras^G12^* ^D^ mice into a tumor-prone *Arf*-null background (*Arf ^−/−^*) **(** **Figure 1** **)**. All *AID-Cre-YFP Kras^G12^* ^D^ *Arf ^−/−^* mice developed rapidly progressive papillomas and by 13 wks, 66% of *AID-Cre-YFP Kras^G12^* ^D^ *Arf ^−/−^* mice (n = 3) developed cutaneous sarcomas **(** **Figure 7A** **)**, while *AID-Cre-YFP Arf ^−/−^* control mice remained disease-free **(** **Figure 7B** **)**. Histopathological sections of spleen from control mice show typical red pulp, white pulp and germinal center structures **(** **Figure 7D** **)**; whereas *AID-Cre-YFP Kras^G12^* ^D^ *Arf ^−/−^* spleen showed defacement of splenic architecture with loss of distinction between red and white pulp and a paucity of germinal centers **(** **Figure 7C** **)**. Sections of sarcomas from *AID-Cre-YFP Kras^G12^* ^D^ *Arf ^−/−^* showed characteristic undifferentiated spindle cells **(** **Figure 7E** **),** consistent with tumors previously described in *Arf*-deficient mice (10). The only abnormalities attributable to B-cells that we identified were small but significant increases in polyclonal antibody responses over time. The gamma protein fraction by SPEP was higher in *AID-Cre-YFP Kras^G12^* ^D^ *Arf ^−/−^* at 12 weeks compared to *AID-Cre-YFP Arf ^−/−^* controls **(Figure S4C)**, but none of the mice developed multiple myeloma or monoclonal gammopathy. *AID-Cre-YFP Kras^G12^* ^D^ *Arf ^−/−^* and *AID-Cre-YFP Kras^G12^* ^D^ *Arf* ^+/−^ mice also showed significant differences in total serum gamma region protein levels between baseline and 12 weeks **(Figure S4A)**. Serum ELISA of antibody subtypes from *AID-Cre-YFP Kras^G12^* ^D^ *Arf ^−/−^*, *AID-Cre-YFP Kras^G12^* ^D^ *Arf ^+/−^*, and control *AID-Cre-YFP Arf ^−/−^* also showed small but significant changes between baseline and 12 weeks in IgM and IgG isosubtypes **(Figure S4B),** perhaps related to infected, fungating papillomas in these mice. Flow cytometric immunophenotyping of bone marrow and splenic mononuclear cells failed to detect the abnormal growth in any B-cell populations in *AID-Cre-YFP Kras^G12^* ^D^ *Arf ^−/−^* mice.

## Discussion

*Kras* is the oncogene most frequently mutated in MM, yet its role in the pathogenesis of the disease has yet to be elucidated. Here, we used a mouse model of activated Kras to directly test the effect of activated Kras in post-germinal center B-cells using two different Cre recombinases reported to be specific to germinal center B-cells. These mice developed T-cell lymphomas, lung adenomas, and sarcomas but no plasma cell tumors despite evidence of activated Kras *in vivo* B-lineage cells. The *Kras* allele was recombined in T-cell lymphomas and lung tumors, suggesting these tumors developed as a consequence of off-target Cre expression. In fact, T-cell lymphomas and lung adenomas have been described in *Kras^G12D^* mice with Cre expressed via adenovirus and *Mx-1* respectively [14], [15], [26].

For malignant transformation in many contexts, activated Ras requires cooperation with additional mutations [27] and we tried several strategies to accelerate disease in *AID-Cre-YFP Kras^G12^* ^D^ mice. Cohorts of *AID-Cre-YFP Kras^G12^* ^D^ mice were subjected to vitamin D deficient chow or sub-lethal radiation or both in an attempt to generate additional mutations and increase the proliferation of pre-malignant B-cells. The combination of vitamin D deficiency and radiation significantly accelerated and worsened the development of skin tumors in *AID-Cre-YFP Kras^G12^* ^D^ mice, but we observed no B-cell phenotype in any of these mice, despite extensive analysis.

Lastly, we engineered mice with a specific cooperating mutation, germinal center expression of Kras^G12D^ in an Arf-null background. The *Ink4a* gene locus encoding both Ink4a and Arf is frequently silenced by hypermethylation in MM [28]–[30] and mutated in some cases of MM ([31] and COSMIC database). Germline mutations in *INK4a* affect predisposition to plasmacytomas in mice [32] and to MM in people [33].

We observed significant acceleration of skin tumors and progression to invasive carcinomas, demonstrating the successful cooperation between the Kras and Arf pathways, but again, these mice failed to demonstrate a significant B-cell phenotype. The development of non-overlapping off-target tumors demonstrates that *Kras^G12D^* can mediate oncogenicity, but germinal center B-cells seem to possess an inherent resistance to its oncogenic effects. We conclude that activation of Kras alone or in the context of Arf pathway inactivation is insufficient to disrupt B-cell homeostasis. These negative data demonstrate that GC B-cells are refractory to mutations which are sufficient to transform other murine tissues, and suggest that distinct tumor suppressor pathways may be active in post-GC B-cells.

The temporal order of acquisition of mutations is likely to be important in the development of some cancers. Observational studies have suggested that Ras activation is a “late event” in myeloma pathogenesis [34]. Ras mutations are significantly less common in patients with monoclonal gammopathy of uncertain significance (MGUS), and are not found in the memory B-cell population of patients with MM, [34] and our data directly supports the model that the temporal order of these events is important to the development of myeloma disease. The lack of a significant B-cell phenotype in our mice is reminiscent of the intrinsic resistance to the effects of Kras^G12D^ displayed by intestinal cells. Intestinal homeostasis is unperturbed in mice by expression of *Kras^G12D^* alone [35], but carcinogenesis occurs with concurrent inactivation of the adenomatous polyposis coli (*APC*) tumor suppressor gene [36]. Mutations in APC do not occur with significant frequency in MM, and it remains unclear what specific mutations cooperate with Ras in myeloma development. In on going work, it will be important to determine the pathways that cooperate with Ras activation to transform germinal center B-cells.

## Methods

### Mouse Strains

*Kras^G12D^* mice [13] (on C57BL/6 background) were crossed to *Cγ1-Cre* knock-in mice [17] (on C57BL/6 background) or *AID-Cre-YFP* transgenic mice [18] (on 129/SvJ × C57BL/6 backgrounds) to obtain double transgenic mice. Triple transgenic mice were created by crossing *Kras^G12D^* mice to *AID-Cre-YFP* and *Arf ^−/−^* or *Arf ^+/−^* mice [20] (on 129/SvJ × C57BL/6 background) **(** **Figure 1** **)**. All mice were routinely observed up to 1 year after birth in a specific pathogen-free facility.

### Ethics Statement

This study was performed in strict accordance with animal use protocols approved by the Washington University Institutional Animal Care and Use Committee (IACUC, protocol number 20120152). Mice were euthanized if they met any early removal criteria (weight loss, lethargy, hunched posture, and/or ruffled coat) to limit suffering, in accordance with NIH-approved institutional animal care guidelines.

### Kras Expression

Raw dataset files of Kras, Hras, and Nras expression in murine B cells was accessible through www.ncbi.nlm.nih.gov/geo (accession GSE4142).

### Stimulation of Germinal Centers

1 mg of (4-hydroxy-3-nitrophenyl) acetyl conjugated chicken gamma globulin (NP-CGG; Biosearch Technologies, Novato, California) was mixed with Freud’s Adjuvant, Complete (Sigma-Aldrich, St. Louis, Missouri) for primary immunization or Freud’s Adjuvant, Incomplete (Sigma-Aldrich) for boosting immunization in 100 ul to inject intraperitoneally at specific time points (**Figure S5)**. Where indicated, *AID-Cre-YFP Kras^G12D^* mice were given a Vitamin D deficient diet (Harlan, Madison, Wisconsin) beginning at 8.5 weeks of age and/or 4 Gy of sub-lethal ionizing radiation at 12 weeks of age.

### Molecular Genotyping of Mouse Strains

For genotyping by PCR, genomic DNA was extracted from tail tissue using Extract-N-Amp Tissue Kit (Sigma-Aldrich). All primer sequences used for genotyping are available upon request. To detect Cre-mediated somatic recombination in bone marrow, spleen, and tumors, genomic DNA was extracted using DNeasy Blood and Tissue Kit (Qiagen, Germantown, Maryland), then amplified using PCR to yield expected 622-bp WT and 650-bp loxP, signifying recombination of the *Kras^G12D^* allele. Mature B cell populations sorted from spleen and bone marrow DNA was extracted using prepGEM Tissue Kit (ZyGEM, New Zealand).

### Ex vivo Class Switch Recombination Assay

Splenocytes from 8–12 week old mice were purified by immunomagnetic depletion of CD43 positive cells (Miltenyi, Auburn, California). CD43 negative splenic naïve B cells were cultured with 15 ng/ml of IL-4 (R&D systems, Minneapolis, Minnesota) and 20 ug/ml of LPS or LPS alone (Sigma-Aldrich) in B cell medium (RPMI-1640 with L-Glutamine (Cellgro, Manassas, Virginia), 1% HEPES, 1% penicillin/streptomycin/amphotericin B, 10% FBS (Hyclone, South Logan, Utah)) at 8×10ˆ5 cells/well of a 6-well plate. Cells were removed from culture at days 0, 3, 5, and 7 for flow cytometric analysis and DNA extraction (Qiagen). On day 5 and 7, cells were split and given 15 ng/ml of IL-4 and 20 ug/ml of LPS or LPS alone.

### Histopathology

Mouse tissues were fixed in 10% neutral buffered formalin for at least 48 hours, dehydrated in an alcohol gradient, cleared in xylene, and infiltrated and embedded in paraffin. Sections were stained for hematoxylin/eosin (H&E).

### Flow Cytometric Analysis

Single cell suspensions of bone marrow and spleen briefly underwent red blood cell lysis.

1×10^∧^6 cells were pre-incubated for 3 minutes on ice with Fc block (CD16/CD32; BD Pharmingen, Franklin Lakes, New Jersey), stained for 25 min on ice with specific antibodies and washed twice in PBS/0.5 M EDTA/0.5 g BSA. The following antibodies used were obtained from BD Pharmingen, unless noted otherwise: FITC-B220 (RA3-6B2), PE-IgM (II/41; eBioscience), APC-IgG1 (X56), AlexaFluor-647 GL7 (eBioscience), PE-CD138 (281-2), PECy7-B220 (RA3-6B2), PE-CD4 (GK1.5), and FITC-CD8. Flow cytometric analysis was performed using FACScan (Becton Dickinson, Franklin Lakes, New Jersey), modified with additional lasers (Cytek Development). FlowJo software (Tree Star, Ashland, Oregon) was used to analyze a minimum of 10,000 events acquired during collection.

### MoFlo

Two 8-week-old *Cγ1-Cre Kras^G12D^* or *Kras AID-Cre* mice were stimulated with 100 ug CGG intraperitoneally and sacrificed 14 days later. Preparation of spleen and bone marrow was previously described. Flow cytometric analysis was performed with the MoFlo single-cell sorter (Becton-Coulter, Brea, California).

### ELISA and SPEP

Total IgA, IgM, IgG, IgG1, IgG2a, IgG2b, and IgG3 levels in serum were measured by enzyme-linked immunosorbent assay (ELISA, Bethyl Laboratories, Inc, Montgomery, TX). Serum protein electrophoresis (SPEP) quantified albumin and globulin proteins from serum using the spife3000 (Helena Laboratories, Beaumont, Texas). Gels were scanned and analyzed using Quickscan2000 software (Helena Laboratories).

## Supporting Information

Figure S1 **Flow cytometry of** ***Cγ1-Cre Kras^G12D^*** **mouse splenocytes undergoing class switch recombination** ***ex vivo*** **.** *Cγ1-Cre Kras^G12D^* mouse splenocytes negatively selected for CD43 and plated in media supplemented with LPS+IL-4. Flow cytometry shows increase of B220+ IgM+ IgG1+ splenocytes at day 3 with LPS+IL-4, compared to day 0.
(TIF)

Figure S2 **Analysis of T-cell lymphomas and lung tumors arising in** ***Cγ1-Cre Kras^G12D^*** **mice.** **A)** Flow cytometry of single cell suspension of naïve *Cγ1-Cre Kras^G12D^* mouse that developed fatal thymus tumor. Lymphoma cells appear to be heterogeneous and composed of CD4+ and double positive CD4/CD8 populations present in both tumor and spleen. Similar results were obtained in 2 additional naïve *Cγ1-Cre Kras^G12D^* mice that developed thymus tumors. **B–E)** Hematoxalin & eosin stains of lung sections from immunized *Cγ1-Cre Kras^G12D^* (**B,C)** and control naïve *Cγ1-Cre* mice **(D,E)** showing incidentally discovered lung tumors. Original magnification, x4 and x40. Scale bar: 500 um and 50 um. **F)** PCR of two lung nodule samples from 2 different *Cγ1-Cre Kras^G12D^* mice show recombination of *Kras^G12D^* locus.
(TIF)

Figure S3 **Subtle changes in immunoglobulin isotype responses in** ***AID-Cre-YFP Kras^G12^*** ^**D**^ **mice detected by enzyme linked immunosorbant assay (ELISA). A)** Total serum gamma region protein levels from *AID-Cre-YFP Kras^G12^* ^D^ and control *Kras^G12^* ^D^ mice calculated from total serum protein multiplied by the percentage of protein in the gamma region of serum protein electrophoresis (SPEP) divided by 100. Results are shown from untreated *AID-Cre-YFP Kras^G12^* ^D^ vs *Kras^G12^* ^D^ mouse cohorts (immunization protocol only; left panel), *AID-Cre-YFP Kras^G12^* ^D^ vs *Kras^G12^* ^D^ cohorts fed vitamin D deficient chow (middle panel) and *AID-Cre-YFP Kras^G12^* ^D^ vs *Kras^G12^* ^D^ cohorts given radiation (right panel). **B)** Serum ELISA of indicated immunoglobulin isotypes of untreated *Kras^G12^* ^D^ and *AID-Cre-YFP Kras^G12^* ^D^ mice. All changes were small in magnitude, but statistically significant differences were noted at baseline in IgM, IgA and IgG3 isotypes, at 9 month IgG2b timepoint and total IgG at endpoint. Serum samples were taken at baseline, prior to immunization with NP-CGG; PPI, post-primary immunization; PBI, post-boosting immunization; 9 mo, 9 month time point; Endpt, endpoint prior to sacrifice. Student’s T-test, *, p<0.05, **, p<0.01, *** p<0.001
(TIF)

Figure S4 ***AID-Cre-YFP Kras^G12^*** ^**D**^ **** ***Arf ^−/−^*** **shows minimal changes in ELISA and serum protein electrophoresis (SPEP). A)** Total gamma region protein levels from serum of *AID-Cre-YFP Kras^G12^* ^D^ *Arf ^−/−^* (DKA, n = 3), *AID-Cre-YFP Kras^G12^* ^D^ *Arf ^+/−^* (DKA^+/−^, n = 2), and control *AID-Cre-YFP Arf ^−/−^* (DA, n = 1) at baseline and 12 weeks, with no immunization. **B)** Serum ELISA of IgM and IgG isotypes of *AID-Cre-YFP Kras^G12^* ^D^ *Arf ^−/−^* (DKA, n = 3), *AID-Cre-YFP Kras^G12^* ^D^ *Arf ^+/−^* (DKA^+/−^, n = 2), and control *AID-Cre-YFP Arf ^−/−^* (DA, n = 1), with statistical significance of IgM isotype of *DKA* and IgG isotype of DKA*^+/−^*. Student’s T-test, *, p<0.05, **, p<0.01, *** p<0.001 **C)** SPEP gel and representative graph showing a low gamma protein of control *AID-Cre-YFP Arf ^−/−^* at 12 weeks, compared to *AID-Cre-YFP Kras^G12^* ^D^ *Arf ^−/−^*.
(TIF)

Figure S5 **Protocol of immunization used in this study.** Mice were injected intraperitoneally with NP-CGG in Freund’s complete adjuvant for primary immunization, followed 4 weeks later by boosting immunization with NP-CGG in Freund’s incomplete adjuvant (arrows). Serum was sampled (arrow heads) at baseline prior to PI, four weeks after primary immunization (post-primary immunization; PPI), four weeks after boosting immunization (post-boosting immunization; PBI), at nine months and prior to sacrifice.
(TIF)
