# Supplementary figures and images for: Germinal Center B-Cells Resist Transformation by Kras Independently of Tumor Suppressor Arf
Source: PLoS One. 2013 Jun 25;8(6):e67941. doi: 10.1371/journal.pone.0067941 (PMC3692489; doi:10.1371/journal.pone.0067941)

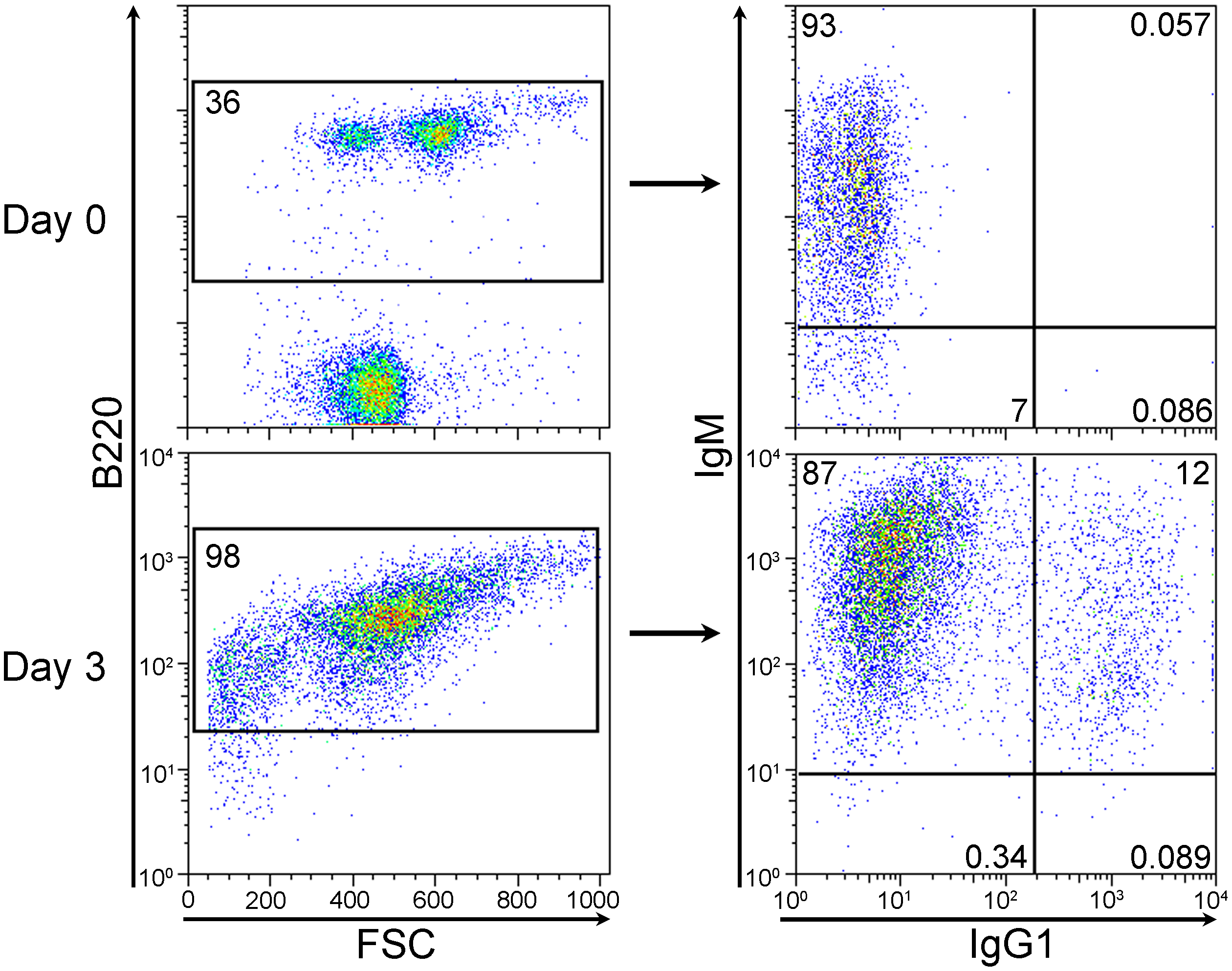

Supplement: Figure S1 — Flow cytometry of Cγ1-Cre KrasG12D mouse splenocytes undergoing class switch recombination ex vivo . Cγ1-Cre KrasG12D mouse splenocytes negatively selected for CD43 and plated in media supplemented with LPS+IL-4. Flow cytometry shows increase of B220+ IgM+ IgG1+ splenocytes at day 3 with LPS+IL-4, compared to day 0. (TIF) [file pone.0067941.s001.tif]

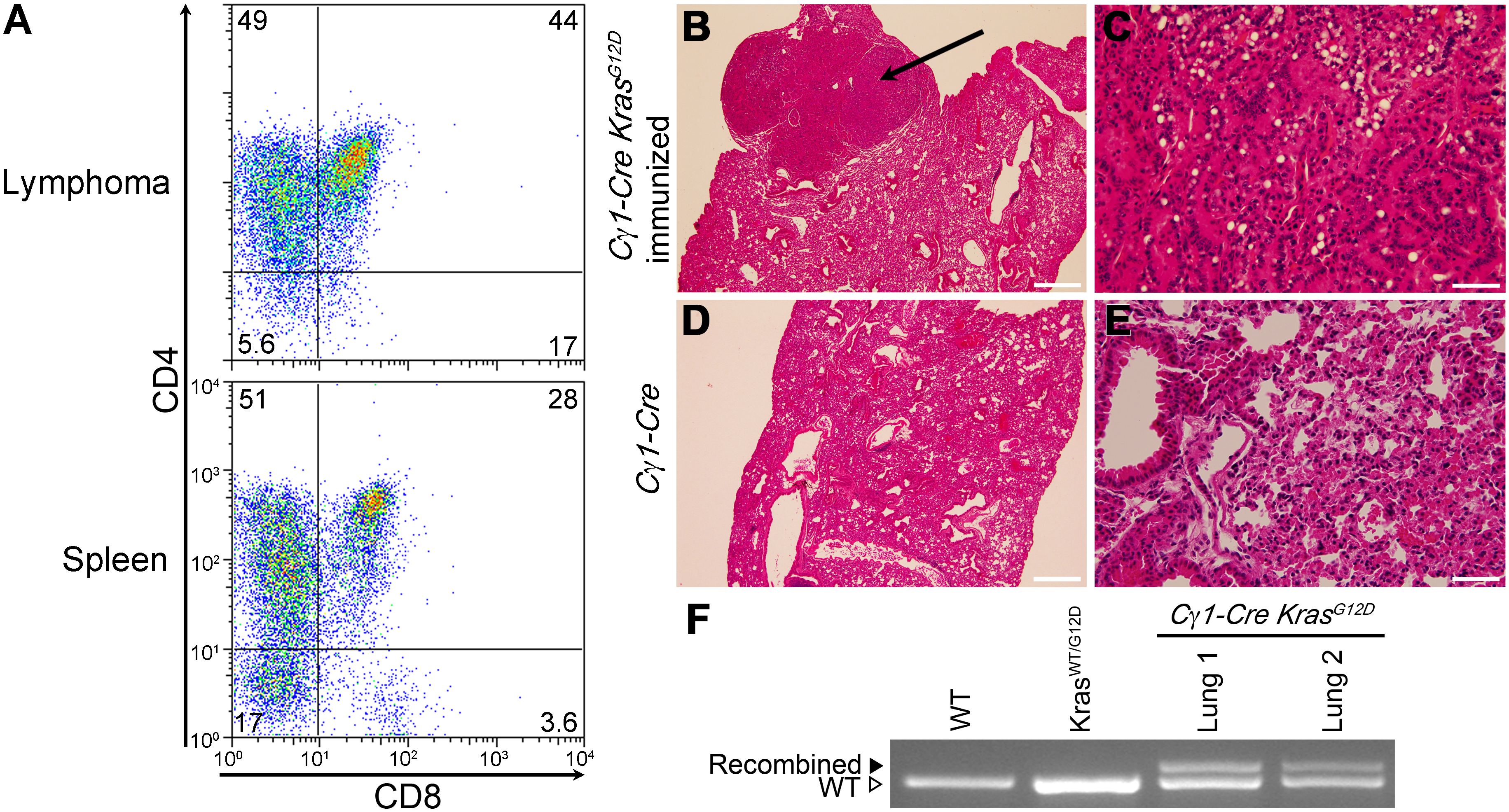

Supplement: Figure S2 — Analysis of T-cell lymphomas and lung tumors arising in Cγ1-Cre KrasG12D mice. A) Flow cytometry of single cell suspension of naïve Cγ1-Cre KrasG12D mouse that developed fatal thymus tumor. Lymphoma cells appear to be heterogeneous and composed of CD4+ and double positive CD4/CD8 populations present in both tumor and spleen. Similar results were obtained in 2 additional naïve Cγ1-Cre KrasG12D mice that developed thymus tumors. B–E) Hematoxalin & eosin stains of lung sections from immunized Cγ1-Cre KrasG12D (B,C) and control naïve Cγ1-Cre mice (D,E) showing incidentally discovered lung tumors. Original magnification, x4 and x40. Scale bar: 500 um and 50 um. F) PCR of two lung nodule samples from 2 different Cγ1-Cre KrasG12D mice show recombination of KrasG12D locus. (TIF) [file pone.0067941.s002.tif]

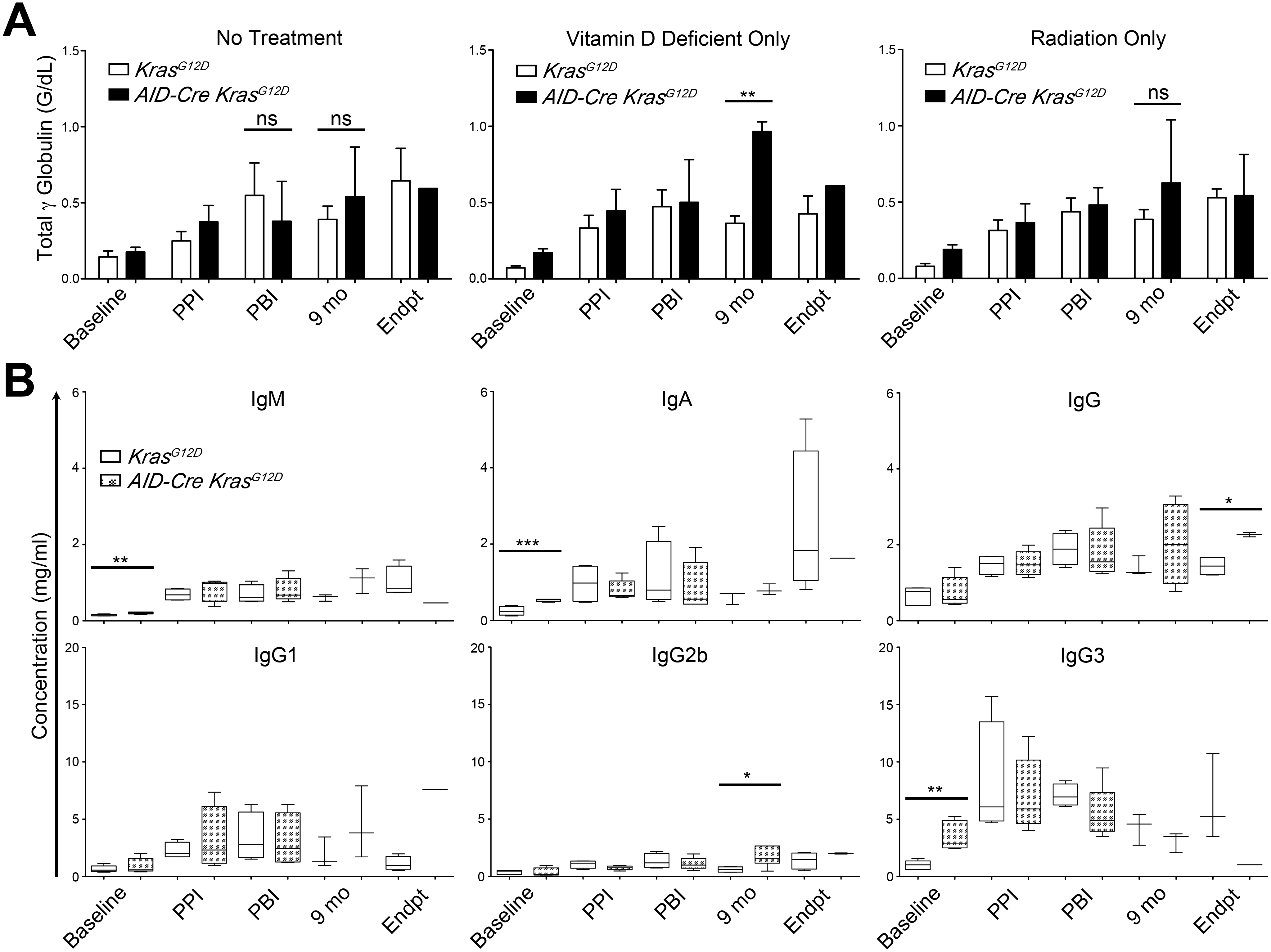

Supplement: Figure S3 — Subtle changes in immunoglobulin isotype responses in AID-Cre-YFP KrasG12 D mice detected by enzyme linked immunosorbant assay (ELISA). A) Total serum gamma region protein levels from AID-Cre-YFP KrasG12 D and control KrasG12 D mice calculated from total serum protein multiplied by the percentage of protein in the gamma region of serum protein electrophoresis (SPEP) divided by 100. Results are shown from untreated AID-Cre-YFP KrasG12 D vs KrasG12 D mouse cohorts (immunization protocol only; left panel), AID-Cre-YFP KrasG12 D vs KrasG12 D cohorts fed vitamin D deficient chow (middle panel) and AID-Cre-YFP KrasG12 D vs KrasG12 D cohorts given radiation (right panel). B) Serum ELISA of indicated immunoglobulin isotypes of untreated KrasG12 D and AID-Cre-YFP KrasG12 D mice. All changes were small in magnitude, but statistically significant differences were noted at baseline in IgM, IgA and IgG3 isotypes, at 9 month IgG2b timepoint and total IgG at endpoint. Serum samples were taken at baseline, prior to immunization with NP-CGG; PPI, post-primary immunization; PBI, post-boosting immunization; 9 mo, 9 month time point; Endpt, endpoint prior to sacrifice. Student’s T-test, *, p<0.05, **, p<0.01, *** p<0.001 (TIF) [file pone.0067941.s003.tif]

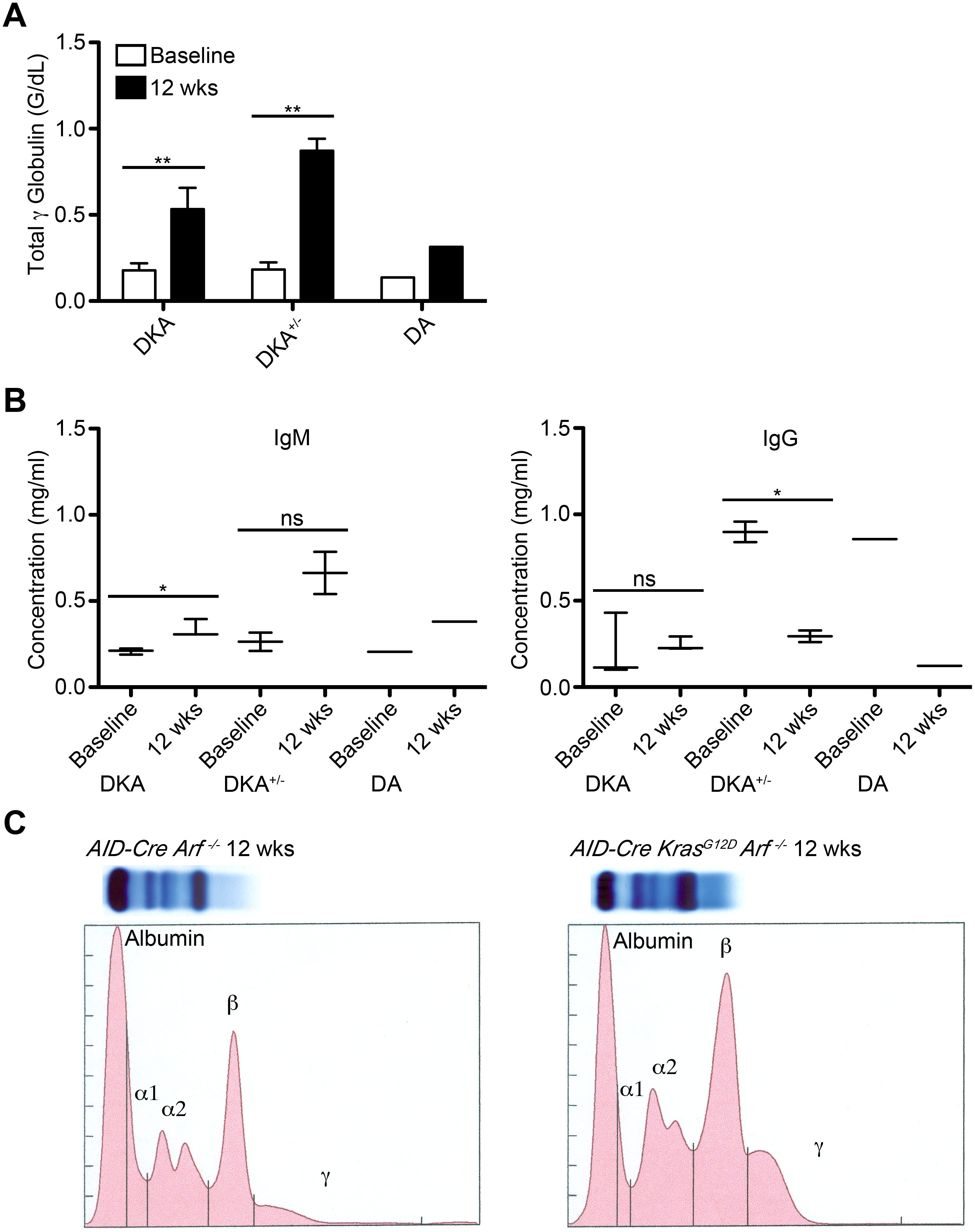

Supplement: Figure S4 — AID-Cre-YFP KrasG12 D Arf −/− shows minimal changes in ELISA and serum protein electrophoresis (SPEP). A) Total gamma region protein levels from serum of AID-Cre-YFP KrasG12 D Arf −/− (DKA, n = 3), AID-Cre-YFP KrasG12 D Arf +/− (DKA+/−, n = 2), and control AID-Cre-YFP Arf −/− (DA, n = 1) at baseline and 12 weeks, with no immunization. B) Serum ELISA of IgM and IgG isotypes of AID-Cre-YFP KrasG12 D Arf −/− (DKA, n = 3), AID-Cre-YFP KrasG12 D Arf +/− (DKA+/−, n = 2), and control AID-Cre-YFP Arf −/− (DA, n = 1), with statistical significance of IgM isotype of DKA and IgG isotype of DKA+/−. Student’s T-test, *, p<0.05, **, p<0.01, *** p<0.001 C) SPEP gel and representative graph showing a low gamma protein of control AID-Cre-YFP Arf −/− at 12 weeks, compared to AID-Cre-YFP KrasG12 D Arf −/−. (TIF) [file pone.0067941.s004.tif]

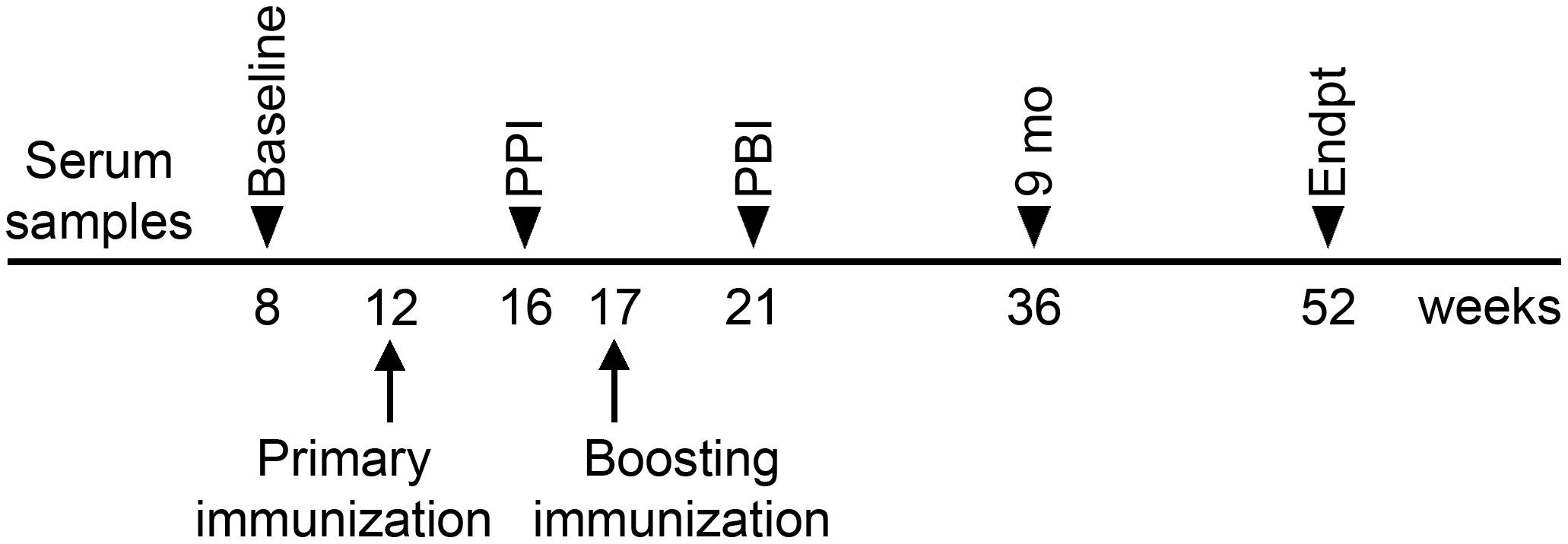

Supplement: Figure S5 — Protocol of immunization used in this study. Mice were injected intraperitoneally with NP-CGG in Freund’s complete adjuvant for primary immunization, followed 4 weeks later by boosting immunization with NP-CGG in Freund’s incomplete adjuvant (arrows). Serum was sampled (arrow heads) at baseline prior to PI, four weeks after primary immunization (post-primary immunization; PPI), four weeks after boosting immunization (post-boosting immunization; PBI), at nine months and prior to sacrifice. (TIF) [file pone.0067941.s005.tif]
